# Supplementary material for: Use of paclitaxel carried in solid lipid nanoparticles to prevent peritoneal fibrosis in rats
Source: PLoS One. 2022 May 6;17(5):e0268197. doi: 10.1371/journal.pone.0268197 (PMC9075628; doi:10.1371/journal.pone.0268197)
Supplement: S1 Table — (DOCX) [file pone.0268197.s001.docx]

**S1 Table.** **Primer sequences used for qRT-PCR.**

| **Primer** | **Sequence of PCR primers** |
| --- | --- |
| **Fibronectin** | Forward 5’-TGACCCAGACTTACGGTGGCA-3’  Reverse 5’-GGAGTAGAAGGTCCTACCGTTGTAGTG-3’ |
| **FSP-1** | Forward 5’-GGCAACGAGGGTGACAAGTT-3’  Reverse 5’-CCCTGGTCAGTAGTCCCTTGA-3’ |
| **VEGF** | Forward 5’-ACTGTGAGCCTTGTTCAGAGCGG3’  Reverse 5’-TCAAGCTGCCTCGCCTTGCA3’ |
| **TGF-β** | Forward 5’-CAACCCGGGTGCTTCCGCAT-3’  Reverse 5’-TGCTCCACCTTGGGCTTGCG -3’ |
| **SMAD3** | Forward 5’-TCAACGGAACTTGGGAATGAG-3’  Reverse 5’-TCACCTCGATCTTGACCTTTTGT-3’ |
| **SMAD7** | Forward 5’-GCGGATCCCTTGGAAAGG-3’  Reverse 5’-GCCACAGCTTCTCCACAGCCA-3’ |
| **Β-actin** | Forward 5’-AGGAGTACGATGAGTCCGGCCC-3’  Reverse 5’-GTAGTGCGGAGCTCTCCTTCA-3’ |
