# Supplementary material for: Use of paclitaxel carried in solid lipid nanoparticles to prevent peritoneal fibrosis in rats
Source: PLoS One. 2022 May 6;17(5):e0268197. doi: 10.1371/journal.pone.0268197 (PMC9075628; doi:10.1371/journal.pone.0268197)
Supplement: Attachment — Submitted filename: Response to Reviewers.docx [file pone.0268197.s002.docx]

Sao Paulo, February 24^th^, 2022.

Prof. Emily Chenette

Editor-in-Chief

*PLOS ONE*

Dear Prof. Chenette

On behalf of our co-authors, we are sending the second revision of our manuscript entitled “Use of paclitaxel carried in solid lipid nanoparticles to prevent peritoneal fibrosis in rats”. Changes made in the manuscript are highlighted in yellow. The replies to the reviewer’s queries are also being enclosed. All the authors agree with this new submission.

Thank you very much for the excellent work of the reviewers and for your kind attention.

Yours sincerely,

Raul C. Maranhão, MD, PhD

Director of the Laboratory of Lipid Metabolism

Heart Institute of the Medical School Hospital.

University of Sao Paulo, Sao Paulo, Brazil

e-mail: [ramarans@usp.br](mailto:ramarans@usp.br)

phone number: 55-11-2661 5574

**Journal Requirements:**

When submitting your revision, we need you to address these additional requirements.

**1.** Please ensure that your manuscript meets PLOS ONE's style requirements, including those for file naming. The PLOS ONE style templates can be found at

https://journals.plos.org/plosone/s/file?id=wjVg/PLOSOne_formatting_sample_main_body.pdf and

https://journals.plos.org/plosone/s/file?id=ba62/PLOSOne_formatting_sample_title_authors_affiliations.pdf

**-** **Ok, the requirements were met.**

**2.** To comply with PLOS ONE submissions requirements, in your Methods section, please provide additional information on the animal research and ensure you have included details on (1) basic housing and breeding, (2) health monitoring, and (3) efforts to alleviate suffering.

**- The additional information on the animal research was included in the Methods section (page 6, lines 121-126).**

**3.** As part of your revision, please complete and submit a copy of the Full ARRIVE 2.0 Guidelines checklist, a document that aims to improve experimental reporting and reproducibility of animal studies for purposes of post-publication data analysis and reproducibility: https://arriveguidelines.org/sites/arrive/files/Author%20Checklist%20-%20Full.pdf (PDF). Please include your completed checklist as a Supporting Information file. Note that if your paper is accepted for publication, this checklist will be published as part of your article.

**- Ok, the requirements were met.**

**4.** Thank you for stating the following financial disclosure:

"This study was supported by the State of Sao Paulo Research Support Foundation (FAPESP, Sao Paulo, Brazil, Grant number 2014/03742-0) and by the National Institute of Science and Technology for Complex Fluids of the Ministry of Science, Technology and Innovation of Brazil."

Please state what role the funders took in the study. If the funders had no role, please state: "The funders had no role in study design, data collection and analysis, decision to publish, or preparation of the manuscript." If this statement is not correct you must amend it as needed. Please include this amended Role of Funder statement in your cover letter; we will change the online submission form on your behalf.

**- The funders had no role in study design, data collection and analysis, decision to publish, or preparation of the manuscript.**

**Reply to the reviewers:**

**Beforehand, we would like to deeply thank both Reviewers for their excellent work that helped us to improve our manuscript.**

**Reviewer #1:**

Thank you for the opportunity to review this work his is a well designed and conducted study investigating the effect of paclitaxel within lipid nanoparticles to mitigate cholhexidine-gluconate induced peritoneal fibrosis.

**1.** While this work has been well designed and conducted the key question relates to whether this has clinical relevance. Specifically, there is limited evidence that chlorhexidine-gluconate is a relevant model of PD-induced peritoneal fibrosis see Vlijm et al 2011 Nephron Exp Nephrol.

**- We are now mentioning other PD animal models and justifying our choice of the chlorhexidine-gluconate model for this study (See Introduction section, with two references included, number 23 and 24, page 5,** **lines 102-111), with further comments in the Discussion section (ref. number 23, page 16, lines 356-359 of the revised manuscript). In respect to** **the Vlijm et al (2011) paper, which is now being quoted (ref. number 30, page 18, line 405-407** **of the revised manuscript).**

**2.** Could the authors comment on why they think that LDE without paclitaxel appears to have 50% as compared with LDE+paclitaxel in mitiigating fibrosis?
**- As showed in Figure 2, the difference observed in the peritoneal thickness of PF group compared with LDE group was not statistically significant. Thus, we assumed that LDE did not have any effect on peritoneal fibrosis, as expected.**

**3.** Please review this sentence
"Conversely, the less expensive, home-based PD procedure, which is more often used in poor countries, has important shortcomings". LMIC (lower and middle income countries) may be a more specific description

**- Ok, done (Introduction section, page 3, line 67).**

**Reviewer #2:**

This study investigates the effect of intraperitoneal Paclitaxel on the formation of peritoneal fibrosis following exposure to chlorhexidine gluconate. The topic is important as a sizeable number of patients undergoing Peritoneal dialysis must go over to hemodialysis due to the loss of ultrafiltration ability of peritoneum secondary to fibrosis. The study design is adequate and the experiment appears well performed as the authors covered many relevant aspects.

**1.** This reviewer would have liked to hear briefly about the potential side effects of Paclitaxel or if the results would have been different if the rats had some sort of kidney impairment.

**- See comments respecting those issues in the Discussion section (page 19, lines 411-424). We also included 4 papers of our previous studies regarding this issue (see reference list, number 31-34, pages 26-27 of the revised manuscript).**
